# Enhancing the driving experience of smart city users based on content delivery framework for intelligent transportation systems

**DOI:** 10.1038/s41598-024-57065-3

**Published:** 2024-03-20

**Authors:** Tariq Alqubaysi, Amr Yousef

**Affiliations:** 1 Department of Civil Engineering, College of Engineering, Northern Border University, Arar, 91431 Saudi Arabia; 2 Electrical Engineering Department, University of Business and Technology, Ar Rawdah, 23435 Jeddah, Saudi Arabia; 3 Engineering Mathematics Department, Alexandria University, El-Naser, Alexandria, 11432 Egypt

**Keywords:** Content delivery networks, Decision-making, Disparity classification, ITS, Smart city, Environmental sciences, Energy science and technology, Engineering

## Abstract

Integration of several communication technologies that facilitate user access contributes to the rapid development of the smart city notion. Intelligent transportation systems (ITS) are introduced as part of smart city development to provide drivers with enhanced communication and information-sharing capabilities. The article introduces a novel ITS content delivery framework (CDF) that addresses communication outage issues. CDF-ITS uses End-to-end decision-making system modelling to examine factors such as communication, content distribution, and vehicle features. A suitable communication slot for vehicular users is determined by processing these characteristics based on outage time and variables. By allocating time-aware communication slots according to the classification of the propagation factor, outage problems may be reduced. End-to-end decision-making is used for classification and vehicle attribute balance, allowing immediate responses to user requests. The experimental outcomes show that the latency of 0.297 s, outage time of 0.0837, distributed messages of 276, and computing complexity of 11.87 are used to assess the proposed framework's efficiency across vehicle density and velocities.

## Introduction

Information and communication technology development and its applications are extended to support roadside information sharing and dissemination through vehicular networks. Conventional vehicular communication networks (VCNs) are connected to external cloud and other interoperable technology providers to provide reliable and service-oriented application support for smart city users^1,2^. Such sophisticated and automated communication support is necessary to manage communication and provide safety assistance for users in roadside driving. Intelligent Transportation Systems (ITS) have emerged under this category by exceeding the communication limits beyond vehicle-to-vehicle (V2V) and vehicle-to-infrastructure (V2I). The boundaries of this intelligent communication network can access distributed information through direct and indirect cloud access. Information sharing and dissemination are facilitated seamlessly through conventional vehicular communication standards and interoperable sensing, actuating and radio units^1,3^. The varying vehicle density and the velocity in the communication region influence the performance of the communicating users for which scalable and information-centric architectures have been designed in recent years. Some common applications of ITS include navigation, user safety and driving assistance, vehicle localization and tracking, etc. These features are exploited by different applications ranging from commercial and healthcare to production and defence industries^4,5^.

ITS couples real-world entities to form a bridge between information and driving users. The design goal of this transportation system is to retain reliability in the communication and data-sharing process to improve the user experience. The issues such as handoff, interference in communication, resource access, and sharing and dissemination are addressed through infrastructures and optimization methods^6,7^. With its interoperable communication features, this intelligent technology assists in performing complex computations and pervasive access to distributed resources. To provide seamless communication across the different service-requesting driving users, information or content dissemination methods are adopted in these vehicular communication networks^7^. The dissemination method differs from the broadcast schemes in that a selected set of neighbours benefit from using the resource dissemination process^8^. In the message dissemination models designed for ITS, the computation and fairness in neighbour selection and content delivery are monitored with the help of connected clouds. Dissemination reliability is ensured by administering replication-free and congestionless transmission aided by the infrastructure units^7,9^.

In the data dissemination models designed for ITS, the reliability of the users is considered by thwarting the adverse effects of vehicle density and velocity. The physical attributes of the vehicles that degrade data dissemination and service compliance in the autonomous driving environment are mitigated in the dissemination methods^8,9^. This is made feasible by integrating different optimization methods and service discovery protocols. Besides, the connected vehicles' smart communication ability and roadside knowledge are exploited in handling content disseminated from multiple sources. The challenging task is to streamline large data queried from distributed and congested environments^10^. The available resources are distributed so the requesting users can meet service demands through vehicle-assisted applications. This context is unanimous for all the connected vehicles that exploit diverse communication technologies on the verge of providing seamless service support for driving users^9–11^.

## Literature review

Sousa et al.^12^ introduced a modular communication architecture for ITS. This architecture's wireless supporting interface integrates communication, operation, and information service models. Using the communication connectivity control unit (CCU), this architecture uses different institutional standards to ease information exchange.

A dissemination point placement optimization problem is focused on by Trullols et al.^13^ to assist ITS communications. On the verge of providing reliable and uninterrupted information exchange between the vehicles, the authors formulate a maximum coverage problem (MCP) for prolonging the communication time between the ITS vehicles.

An integrated data exchange platform (IDEP) is designed in^14^ for ITS. IDEP framework operates through the vehicles' different physical and communication layers to provide reliable data exchange between the driving users. The design goal of this framework is to provide scalable and secure data exchange, suppressing the issues due to the varying density and velocity of the vehicles through connected network assistance.

Big data-assisted V2X communication (BDAC) is designed by An and Wu^15^ to augment the performance of smart transportation systems. The area density and neighbour velocity are estimated based on the stored road-side information in the distributed cloud. This estimation helps to classify vehicles based on data handling rate, improving packet dissemination and reducing delay.

Fan et al.^16^ proposed a replication-based distributed randomized algorithm (R-DRA) for improving data dissemination in connected vehicles. The data to be disseminated is allocated for multiple other infrastructure units to reduce overloaded network resource exploitations. This algorithm’s communication complexity and convergence are reduced by assigning an allocated data exchange process.

A cross-layer design for reliable data dissemination in vehicular ad-hoc networks is proposed by Duan et al.^17^. This cross-layer design is modelled using interference-aware power-control (IAPC) optimization. This optimization helps improve the network’s throughput using a linear programming model. This cross-layer design maximizes network throughput by reducing service time and loss.

An efficient data dissemination protocol (EDDP) proposed by Chaqfeh et al.^18^ spotlights the improvement of data delivery and the reduction of overhead and delay in urban vehicle communications. EDDP relies on the local information shared by the infrastructure units to analyze the feasible conditions for data dissemination. Based on the location and attributes of the received messages, broadcast storm issues are mitigated in this protocol to achieve better performance.

Zhou et al.^19^ analyzed the feasibility of assimilating physical and social layers for data dissemination in the Internet of Vehicles. The connectivity issues in the physical layer based on distance are analyzed using the Weiner process. Bayesian non-parametric learning model is employed in the social layer for content assortment and dissemination. These altering processes are assimilated to improve the QoS of the connected vehicle data dissemination and big data sharing instances.

The authors in^20^ exploited named data networking (NDN) architecture to improve data dissemination efficiency in VANETs. The message’s description and content focus on improving the delivery rate rather than the connected host units using this architecture. This architecture is modelled around stand-alone RSUs (SA-RSUs) to reduce re-transmission instances, handle varying network loads, and improve delivery rate with controlled delay.

An on-demand member-centric routing (OMR) protocol was proposed by Huang et al.^21^ to improve the data dissemination of IoV-assisted applications. This protocol uses a reactive member-centric mechanism (RMCM) using different dissemination strategies. The strategies focus on balancing network load between the communicating vehicle pairs, employing a helper-disjoint algorithm. The advantage of this protocol is the amalgamation of multi-source traffic, reducing the collision probability.

To leverage message dissemination in VANETs, Nguyen et al.^22^ proposed a store-carry-forward (SCF) mechanism. This mechanism addresses the issues due to network segregation and frequent broadcasts in handling data. The physical attributes of the vehicles, such as heading direction, speed, and location, and the communication attribute of warning beacons are analyzed to model the reliability of data dissemination in this mechanism.

The design of distance-based relay selection for vehicular message dissemination is introduced by Cao et al.^23^. Relay selection is carried out with the help of exponent-based partitioning broadcast protocol [check ref]. The mini-black-burst mechanism employed in this selection scheme helps mitigate the broadcast storm problem, achieving better message dissemination and flow rate.

The proposal in^24^ discusses a reliable message dissemination scheme using smart devices for the Internet of Things (IoT). A low-cost code dissemination model that enables opportunistic vehicular communications is proposed for this purpose. Greedy deployment for better coverage and optimal code selection aids improved message dissemination under controlled delay in the IoT vehicular environment.

Baiocchi^25^ analyzed message dissemination protocols employed for inter-vehicle communications. Message covering distance and delivery delay in timer-based dissemination protocols for highway VANETs is analyzed in this proposal. With the help of Poisson distribution and spatial representations, the dissemination protocol is analyzed for its decay rate, hop length and delay, and dissemination speed.

Based on the survey, there are several issues with existing models in attaining less latency, outage time, distributed messages, and computing complexity. The article introduces a novel ITS content delivery framework (CDF) that addresses communication outage issues.

## Content delivery framework (CDF) for ITS

This article discusses CDF-ITS to improve the driving experience of end users. Providing seamless access to resources and service distribution in an ad-hoc manner helps improve the vehicle users' driving experience. Besides in-time delivery and prolonged communication, it helps to meet the user requirements. For this purpose, contact dissemination and data sharing in the mobile environment has to be optimized. Vehicle characteristics and communication interferences degrade the quality of data dissemination and content delivery. The content marks up different data classes, from sensor information to multimedia shared through infrastructure units and wireless channels.

### System architecture

The architecture of ITS is modelled with $V = \left\{ {v_{1} ,v_{2} ,v_{3} , \ldots .v_{n} } \right\}$ set of vehicles connected through wireless channels $c = \left\{ {c_{1} ,c_{2} ,c_{3} , \ldots .c_{n} } \right\}$. In that case, a graph $G = \left( {V,C} \right)$ denote the connected vehicular environment.

**Figure 1:**
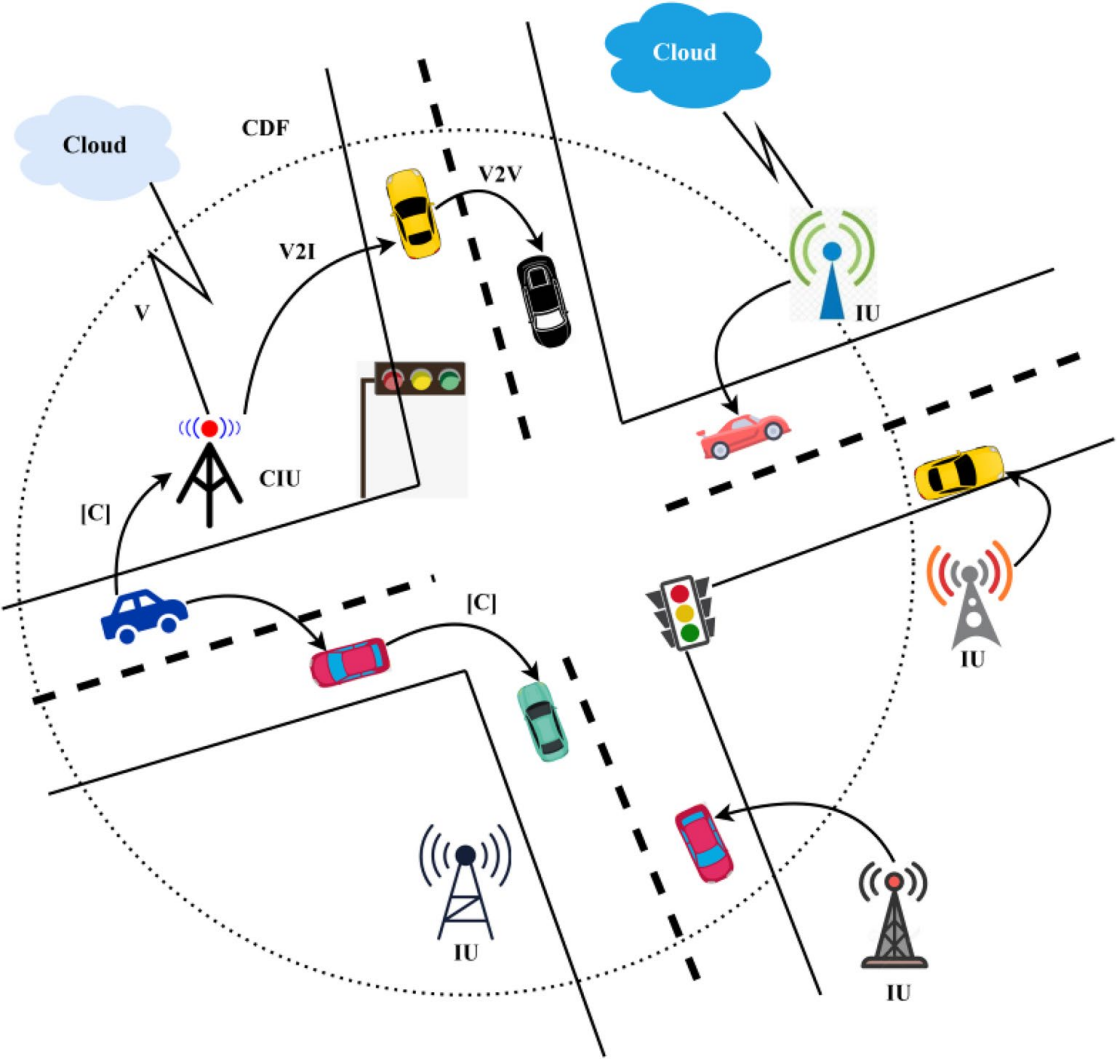
System architecture.

The vehicles can gain information through a cloud platform by exploiting its smart communication ability. According to the conventional vehicular communication system, there are two modes of communication: vehicle-to-vehicle (V2V) and vehicle-to-infrastructure (V21). A vehicle communicates with the cloud using V21. An illustration of the system architecture is presented in Fig. 1. The infrastructure units (IU) assist V2I communications in the architecture using the channels represented as [C].

### Communication model

As mentioned earlier, vehicle communication is performed using wireless channels. The available data/content is disseminated over the active channels as a whole or distributed manner. In conventional vehicular communication, the information is accessed and acquired from multiple sources through different infrastructure units. This helps to adapt the data dissemination process over the varying vehicle speeds and density. Instead, handling multiple data streams/disseminating large volumes of data through a common channel results in internal congestion. If the vehicle experiences an outage, all the data is lost or fails. Therefore, the communication is split into slots for content handling. The source for the originating content adopts time slots for disseminating information successfully. The number of slots (S) required for content dissemination is given by Eq. (1)

1 $$\left. \begin{gathered} S = x \times d_{h} \hfill \\ where, \hfill \\ d_{h} = \sum (d_{m} ,d_{p} )\;and \hfill \\ x = v\left[ {1 - \frac{{\min \left( {D,R} \right)}}{R}} \right] \hfill \\ \end{gathered} \right\}$$

Here, $x$ is the slot number, $d_{h}$ is the delay experienced in one hop, $d_{m}$ and $d_{p}$ is the medium access and propagation delay, respectively. The variables $D$ and $R$ are used to denote the distance between the vehicles and the radio range, correspondingly.

The above slots are allocated to reduce the latency in information dissemination. Therefore, the objective of the CDF is given by Eq. (2)

2 $$\left. {\begin{array}{*{20}l} {minimize:d_{l} } \hfill \\ {such \;that,} \hfill \\ {1 - \mathop \prod \limits_{i = 1}^{V} (1 - \rho \left\{ {d_{h} \le d_{w} } \right\}\forall {\text{max}}\left\{ \Delta \right\}} \hfill \\ \end{array} } \right\}$$

The objective represented in Eq. (2) demands minimum latency $d_{l}$ provided maximum dissemination delivery $\left( \Delta \right)$ is achieved. And for all the allocated slots, the waiting time $\left( {d_{w} } \right)$ is less than or equal to the one-hop delay. Similarly, this condition needs to be achieved until the probability $\rho \left\{ {d_{h} \le d_{w} } \right\}$ is true. In this objective, the outage time $\left( {d_{o} } \right)$ is augmented with $d_{w}$ such that $d_{w} = d_{q} + d_{o}$, where $d_{q}$ denotes the queuing time. The decision-making process acts upon different criteria to reduce $d_{q}$ and $d_{o}$. The decision-making process aims to assign communication slots for the active vehicles irrespective of the $d_{q}$ and $d_{o}$. The expected data dissemination to the one-hop neighbor $\left( {\varphi_{s} } \right)$ is computed as

3 $$\varphi_{s} = 1 - \frac{1}{\left| V \right|}\mathop \sum \limits_{i = 1}^{V} \mathop \sum \limits_{j = 1}^{{v_{p} }} \frac{{C_{d}^{j} /C_{r}^{i} }}{{(v_{p} )_{ij} }}$$

Here, $v_{p}$ is the number of path vehicles, $C_{d}^{j}$ and $C_{r}^{i}$ are the content disseminated through the path vehicles and content dissemination requests through the available vehicles. Maximizing the above relies on the two factors $d_{q}$ and $d_{0}$ for which the decision-making is incorporated. The decision-making process is split for the above metrics based on the density and velocity of the vehicles in the dissemination range. In the following subsection, the decision-making in the independent analysis of $d_{q}$ and $d_{o}$ is discussed in detail.

### Impact of vehicle density

**Figure 2:**
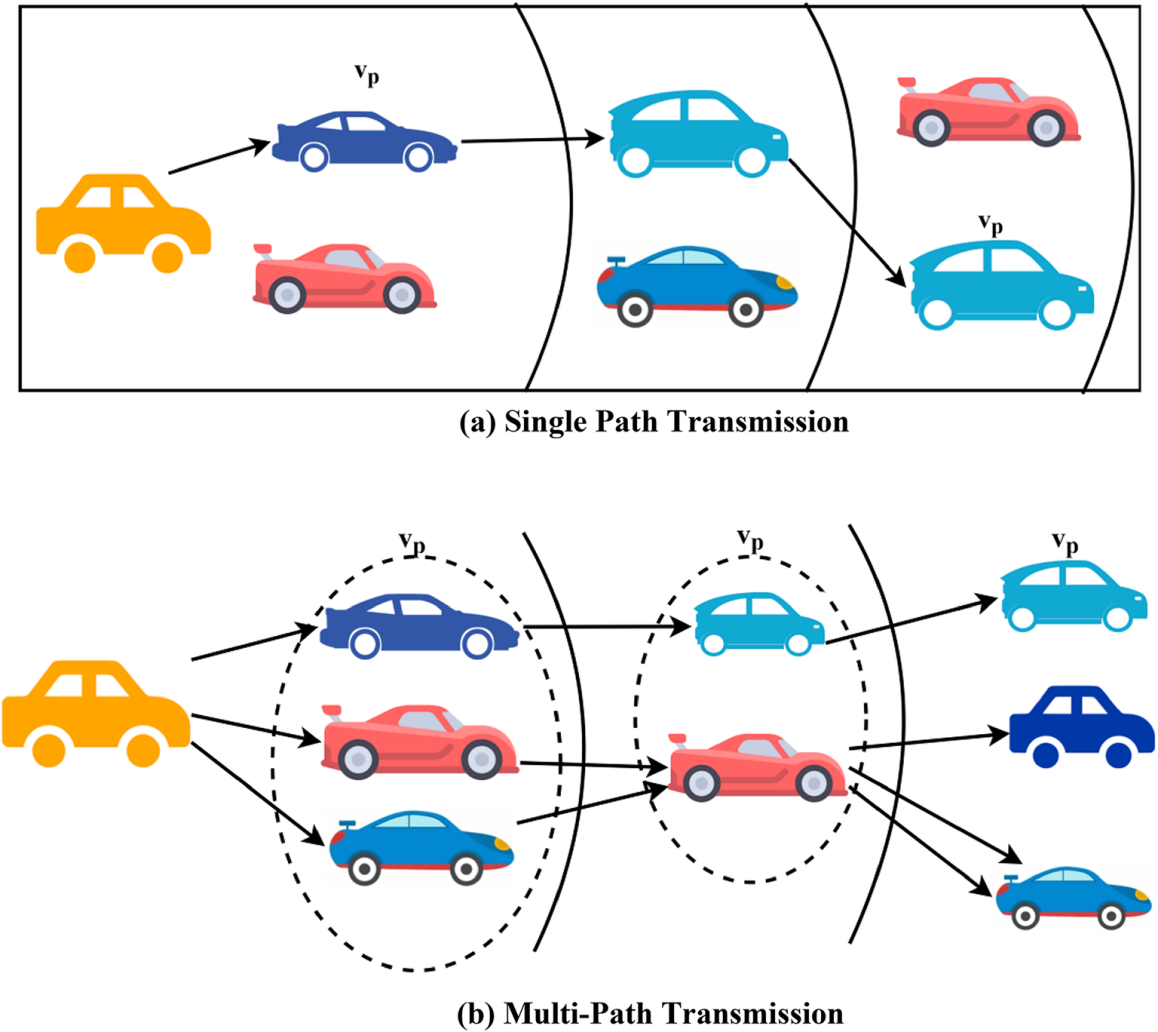
(**a**) Single path transmission. (**b**) Multi-path transmission.

The vehicles’ density is subject to change with the connectivity and range of the transmitting source. Based on the range of the vehicle and the distance between the source and receiving vehicles, the density of the vehicles is split into different regions, as mentioned in Fig. 2a and b

The vehicles' density and content requesters’ availability decide the transmission as single or multi-path. The decision-making level $\left( {f_{L} } \right)$ is computed as

4 $$\left.\begin{array}{c}{f}_{L}=\frac{|\bigcap_{\begin{array}{c}\Delta \\ {v}_{p\in V}\end{array}}|}{\left|V\right|}\\ {f}_{L}=\frac{\bigcap_{{v}_{p\in V}}\frac{{c}_{d}}{{c}_{r}}}{|V|}\end{array}\right\}$$

In the above equation, content delivery is the major concern such that the path vehicles $\left( j \right)$ must $\in$ the set of $i$ (as in Eq. (3)). This means the dissemination process occurs with existing vehicle density for all $v_{p} \in V$. The factor $f_{L}$ denotes the maximum accuracy of the decision-making process. This is valid until the split region owns a constant density (as in Fig. 2a). On the other hand, due to changes in velocity and distance, $R$ of the vehicle is capable of accommodating multiple neighbours where the same $f_{L}$ cannot be sustained throughout $d_{h}$. In a single path dissemination, $S$ is mapped with one-neighbour in the following $R,$ where $\frac{{c_{d} }}{{c_{r} }}$ is the neighbour's determining factor. The variables $\Delta$ and $\left( {c_{d} /c_{r} } \right)$ are used to compute the same message delivery factor provided the density and $d_{q}$ varies for the allocated slots.

For a content dissemination process, as in Fig. 2a, the $v_{p}$ are swapped if a disparity $\Delta$ is observed in $S$ and the next neighbour is replaced. Interestingly, the same $S$ is allocated for the new vehicle satisfying $v_{p} \in V$ condition. The disparity in $f_{L}$(i.e.) $\left( \gamma \right)$ is computed as

5 $$\gamma \left( {f_{L} ,\Delta = \frac{{\left| {\mathop \cap \limits_{{v_{p} \in v}} \left( {1 - \Delta } \right)} \right|}}{\left| V \right|}} \right. = \frac{{\left| {\mathop \cap \limits_{{v_{p} \in v}} \frac{{c_{r} - c_{d} }}{{c_{r} }}} \right|}}{\left| V \right|}$$

This disparity is computed if there is a change in $\Delta$ is $S$. Therefore, $\gamma \left( {f_{L} ,\Delta } \right)$ as in Eq. (5) is the threshold for retaining $v_{p}$ in the dissemination process. On the other hand, for a multi-path, the slots are allocated based on the $v_{p}$ that is observed in the $R$. Therefore, $S = V \in R$ is the allocated slot for content dissemination. The disparity in content dissemination (as in Fig. 2b) varies for the available vehicles in each region of the one-hop vehicles. The allocated slots must ensure sequential dissemination $d_{h}$ such that is the necessary condition.

6 $$\left. {\begin{array}{*{20}c} {\Delta_{1} = \Delta_{2} = \cdots \Delta_{n} , \forall \left( {x \times d_{h} } \right) = S} \\ {such \;that\; d_{q} = d_{h} d_{w} = 0} \\ \end{array} } \right\}$$

Hence, the disparity is validated as follows:

If any of the $v_{p}$ in the above dissemination process does not satisfy Eq. (6), then

7 $$\left. {\begin{array}{*{20}l} {\gamma \left( {f_{L} ,\Delta } \right) = \left| {f_{L} } \right| - |f_{L} ,\Delta )\left| { \ge } \right|f_{L} *\left. {\left( {1 - \varphi_{s} } \right)} \right|} \hfill \\ { = \left| {f_{L} } \right| \cap \left| {\gamma \left( {f_{L} ,\Delta } \right)} \right| \in v_{p} \;in \;V} \hfill \\ { = \left| {f_{L} } \right|\left( {| \cap \gamma \left( {f_{L} ,\Delta } \right)} \right) \in v_{p} } \hfill \\ \end{array} } \right\}$$

**Figure 3:**
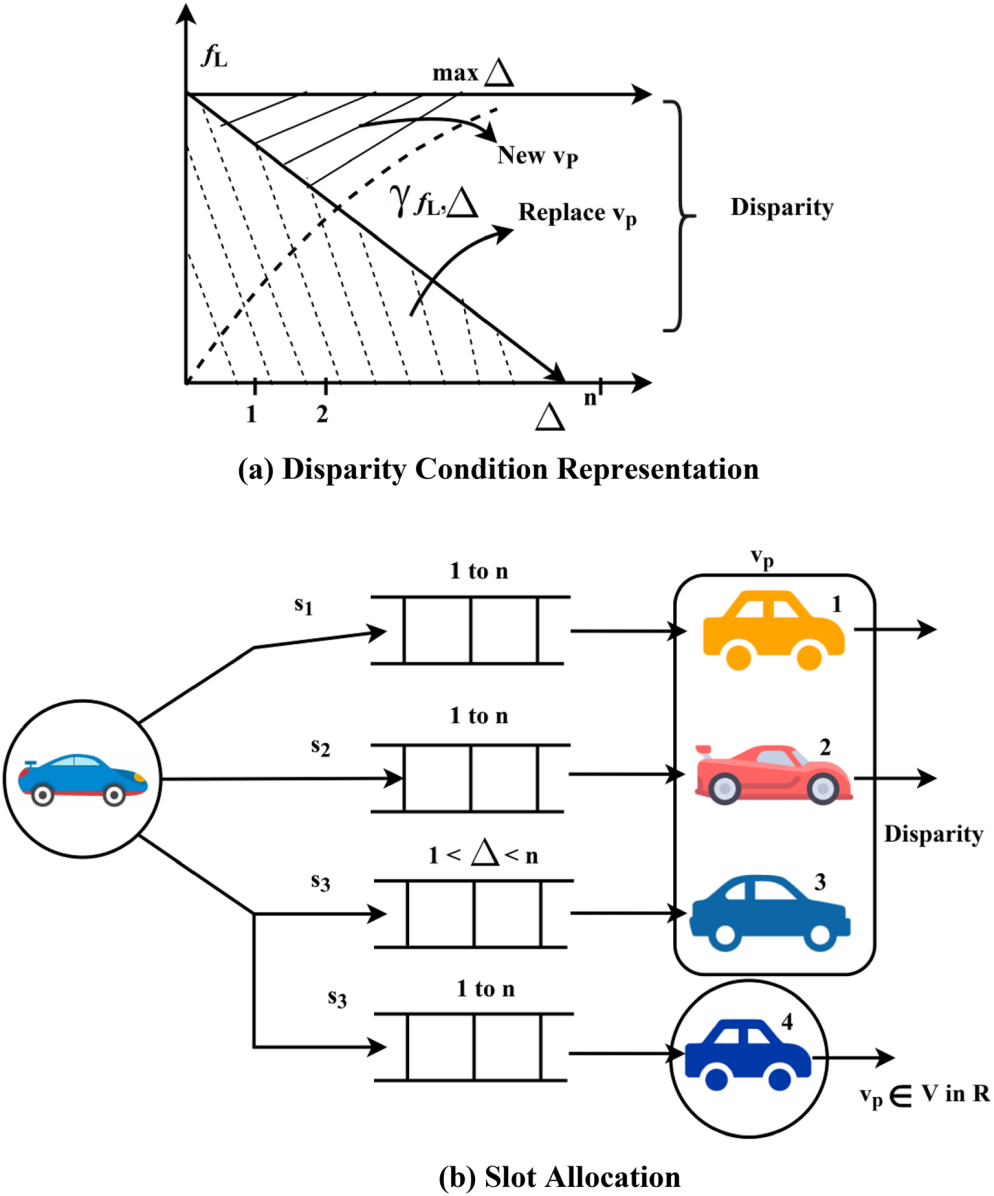
(**a**) Disparity condition representation. (**b**) Slot allocation.

In Eq. (7), the disparity vehicle is identified in the available split regions, and that is replaced by the new vehicle $v_{p}$. The variation in $\Delta$ for $n$ dissemination instances is repeated in the same manner until the condition in Eq. (6) is satisfied. In Fig. 3a and b, the disparity condition and slot allocation process for the scenario in Fig. 2b is illustrated.

As represented in Fig. 3a, the vehicles classified under $\left[ {1 - \gamma \left( {f_{L} ,\Delta } \right)} \right]$ or $\gamma \left( {f_{L} ,\Delta } \right)$ are said to be disparity. The slots allocated for these vehicles are re-allocated for a new $v_{p}$ in the same $R$ such that $v_{p} \in V$. The allocation is valid until Eq. (6) is satisfied, by which ${\text{max}}\left\{ \Delta \right\}$ is feasible in all $S$. Contrarily, if Eq. (7) is achieved in any $v_{p}$, then slot re-allocation takes place.

### Impact of vehicle velocity

The position of a $v_{p}$ is unpredictable with changes in the velocity of the vehicle. Therefore, the $C$ between the vehicles does not sustain throughout the dissemination. Unreachable or interrupting dissemination results in an outage. The outage time needs to be suppressed or mitigated to ensure $\min \left\{ {dw} \right\}$ and ${\text{max}}\left\{ {\varphi_{s} } \right\}$. As the outage is minimized, the expected dissemination is $\varphi_{s}$ and not $\Delta$. Therefore, the decision-making is intended to retain $\varphi_{s}$ not below $\left[ {1 - \gamma \left( {f_{L} ,\Delta } \right)} \right]$. Besides, the decision must be abrupt to leverage dissemination from $\varphi_{s} to \Delta$ such that $t_{o}$ holds only for a while. Therefore, the objective of $d_{h} \le d_{w}$(as in Eq. (2) is focused in this part to reduce the outage time $\left( {t_{o} } \right)$. The $t_{o}$ is computed as

8 $$t_{o} = P\left\{ {\gamma \left( {f_{L} ,\Delta } \right) > \varphi_{s} } \right\}*d_{h}$$

In Eq. (8), the probability of the fall in $\Delta$ is computed for the interval, where the $v_{p}$ is said to be experienced $t_{o}$. In the proposed framework, velocity is considered as an influencing factor for $t_{o}$. If the velocity of the vehicle $\left( {V_{v} } \right)$ is incongruent with the $v_{p}$ for a time $d_{h} = d_{q}$, $d_{w} = 0$, then $t_{o}$ is less. This is because the dissemination time ranges between $(d_{h} \times v\_p$) to $\left[ {S*d_{h} + d_{w} } \right)]$ for the connected $v_{p}$. The first time computed (i.e.) $\left( {d_{h} \times v_{p} } \right)$ is an ideal scenario where the neighbours of $v_{p}$ follows single-path dissemination. The decision for the

9a $$\left. {\begin{array}{*{20}c} {f_{L} \left( {d_{h} } \right) = \frac{{\left| {\mathop \cap \limits_{{v_{p} \in p}} \left( {d_{h} *v_{p} } \right)} \right|}}{\left| V \right|}, \;if \;single\; path} \\ {\begin{array}{*{20}c} {such\; that} \\ {\gamma \left( {f_{L} ,\Delta } \right) = 0\;in \left( {d_{h} \times v_{p} } \right) - \left( {d_{h} } \right)\;interval} \\ \end{array} } \\ \end{array} } \right\}$$

9b $$\left. {\begin{array}{*{20}c} {\begin{array}{*{20}c} {f_{L} \left( {d_{h} } \right) = \frac{{\left| {\mathop \cap \limits_{{v_{p} \in p}} \left( {d_{h} + d_{w} } \right)*S} \right|}}{\left| V \right|},\; if\; multipath} \\ {such \;that } \\ \end{array} } \\ {\gamma \left( {f_{L} ,\Delta } \right) = \varphi_{s} \; in \;any \Delta_{i} \ne \Delta_{i + 1} } \\ \end{array} } \right\}$$

The disparity here is based on successful transmissions experienced in the time range. The successful transmission here notifies the $\varphi_{s}$ or $\Delta$ experienced in $[S*\left( {d_{h} + d_{w} } \right)$ or $\left( {d_{h} \times v_{p} } \right)$. Leaving out $\Delta$ in $\left( {d_{h} \times v_{p} } \right)$ time, the decision-making is considered for $\left[ {s*\left( {d_{h} + d_{w} } \right)} \right]$ time as the chances of $\Delta_{i} \ne \Delta_{i + 1}$ is high, which degrades the dissemination process. The $v_{p}$ is selected based on the capacity utilized in $S$. The capacity ($\tau )$ of a $v_{p}$ is defined as

10 $$\tau = r_{d} *\left[ {1 - \gamma \left( {f_{L} ,\Delta } \right)} \right] \times \left( {d_{h} + d_{w} } \right)$$

Based on Eq. (10), the disparity in multi-path dissemination due to outage [for Eq. (9b)] is represented as

**Figure 4:**
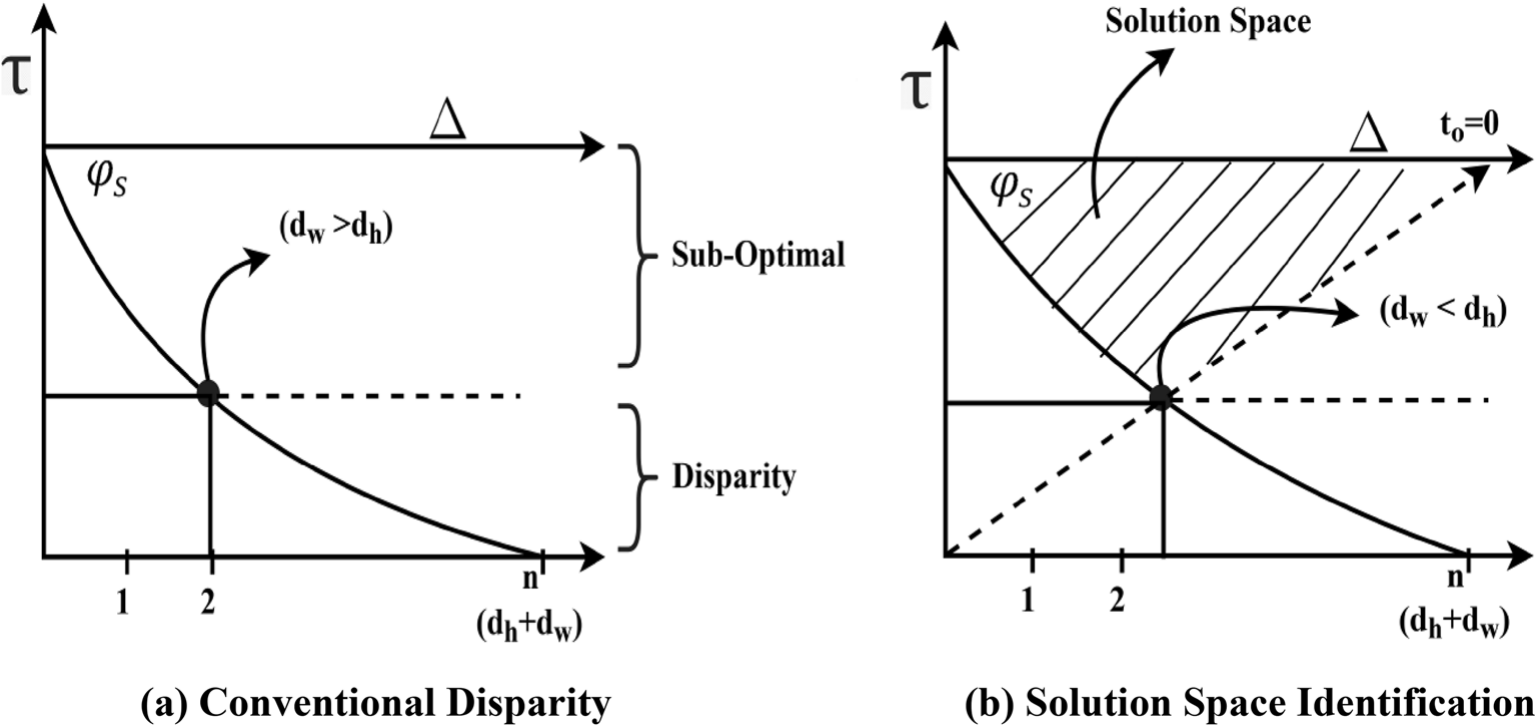
(**a**) Conventional disparity. (**b**) Solution space identification.

This case, represented in Fig. 4a, denotes the conventional disparity of $$d_{o}$$ increases in $$\left( {d_{h} + d_{w} } \right)$$. On the other hand, the case of deriving sub-optimal and ideal solutions with the estimation of disparity $$\left( {d_{w} > d_{h} } \right)$$ point is as presented in Fig. 4b. The deriving point of $$(d_{w} < d_{h} )$$ ensures a sub-optimal solution, whereas if $$d_{o} = 0$$, then it is an ideal case; therefore, the time required for balancing the outage is $$\left( {d_{h} - d_{w} } \right) > 0$$ or $$d_{h} > d_{w}$$. Therefore, if $$d_{h} > d_{w}$$, then $$\left( {d_{h} + d_{w} } \right)$$ (as $$d_{w} = 0)$$ is the time where $$\varphi_{s}$$ is achieved. Therefore, the capacity of the $$v_{p}$$ is $$r_{d} *\left[ {\frac{{c_{d} }}{{c_{r} }}} \right]*d_{h}$$. This capacity is to be retained by the $$v_{p}$$ in the time interval $$\left[ {\left( {d_{h} + d_{q} } \right), \left( {d_{h} *v_{p} } \right)} \right]$$ where $$t_{o}$$ is minimum. Therefore, the disparity point is $$\frac{{\left| {\mathop \cap \limits_{{v_{p} \in V}} } \right.\left( {d_{h} + d_{q} } \right)S}}{\left| V \right|}$$, if the vehicle is selected in $$(d_{w} < d_{h} )$$ to $$d_{o} = 0$$ region [Solution space, refer to Fig. 4b]. The vehicle selected in this solution space ensures less $$d_{o}$$, provided $$d_{h} < d_{w}$$ where $$d_{h} = d_{w}$$ is the disparity point. This helps mitigate outage impact due to the varying vehicle speed.

## Simulation environment

The performance of the proposed CDF-ITS is verified using simulations carried out using a network simulator and SUMO traffic modelling and metrics such as latency, outage time, distributed messages and computing complexity. The dissemination framework illustrated in Fig. 1 is used to construct the ITS scenario with external distributed networks. The scenario consists of 200 vehicles connected with a location service providing a cloud network. The location information of the vehicles and their last access positions are stored in the cloud, and the space allocated for the cloud is 250 GB. With the help of 32 IU, the dissemination process is modelled across the vehicles in single and multi-path. In Table 1, the detailed simulation environment is specified.

**Table 1:** Simulation environment.

Simulation setup	Values
Vehicles	40–200
Velocity	50–100 km/h
IU	32
Channel bandwidth	5 Mbps
Cloud storage	250 Gb
Cloud resources	4
Dissemination rate	10 messages/s

The above-tabulated environment is used for analyzing the performance of CDF-ITS using the metrics dissemination latency, outage time, dissemination messages, and dissemination complexity for the varying vehicle density and velocity. The case of decision-making using the disparity model plays a vital role in classifying the optimal and disparity solutions in this content dissemination framework. For a comparative study, the existing methods EDDP^18^, R-DRA^16^, and BDAC^15^ are considered for the changing velocity and density. In this analysis, the velocity is kept constant for the varying density of vehicles, and vice-versa.

## Results and discussion

### Analysis based on varying vehicle density

As the density of the vehicles varies, the connectivity and $c$ as in $G$ is affected. It causes both reliable and adversary effects on the content dissemination framework. High is the density of the vehicles, and high is the $v_{p}$ availability and the dissemination rate. The impact of vehicle density on the metrics dissemination latency, outage time, messages and complexity is analyzed in this section.

**Figure 5:**
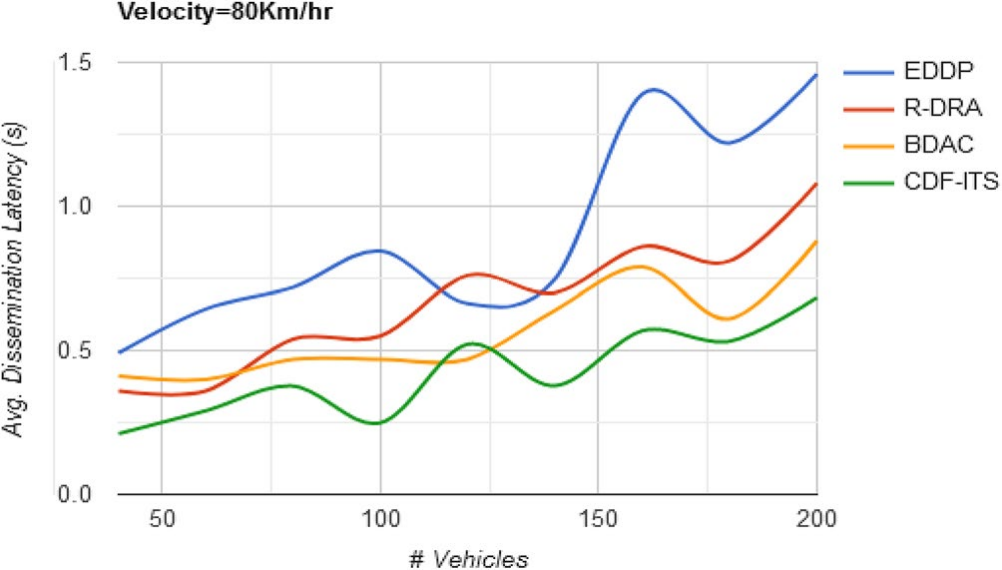
Avg. dissemination latency versus vehicles.

Message dissemination concerning the varying vehicle density (Fig. 5) is retained by retaining the available $$c$$. This is pursued in single and multi-path data dissemination to satisfy the objective of $${\text{max}}\left\{ \Delta \right\}$$. This condition is satisfied by differencing $$f_{L}$$ and $$\gamma \left( {f_{L} , \Delta } \right)$$ in the allocated $$S$$. The $$v_{p}$$ classified based on dissemination rate is allocated with sequential or alternating slots for content delivery. Therefore, the latency observed here (irrespective of the velocity pf the vehicle) is $$\left( {v_{p} *d_{h} } \right)$$ in a single path and $$d_{h} + d_{q} )$$ for multi-path dissemination. The maximum time bounds for 1 to $$n$$ dissemination interval lies within the range as mentioned above that is less until $$(d_{h} < d_{w} )$$. Similarly, the outage time is lost by replacing the $$v_{p}$$ based on disparity condition (w.r.t. density). In the disparity condition defined, the solution space for replacing $$v_{p}$$ is identified in $$\gamma \left( {f_{L} ,\Delta } \right)$$ observations and the optimal occurrence is $${\text{max}}\left\{ \Delta \right\}$$ or $$\left[ {1 - \gamma \left( {f_{L} ,\Delta } \right)} \right]$$ is case of multi-path content dissemination.

**Figure 6:**
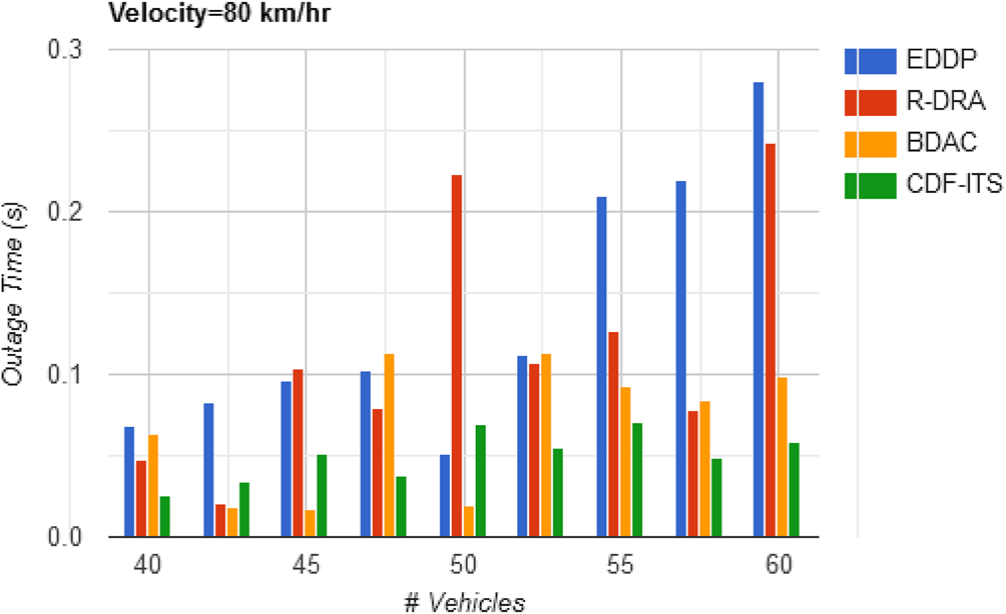
Outage time versus vehicles.

Therefore, the outage (w.r.to density) is observed below $\gamma \left( {f_{L} ,\Delta } \right)$ time where $\Delta_{i} \ne \Delta_{i + 1}$ as estimated using Eq. (8). The range of $v_{p}$ achieving ${\text{max}}\left\{ \Delta \right\}$ is always separated from the $v_{p}$ that does not meet the objective, so the replacement is done easily, reducing the outage time (refer to Fig. 6).

**Figure 7:**
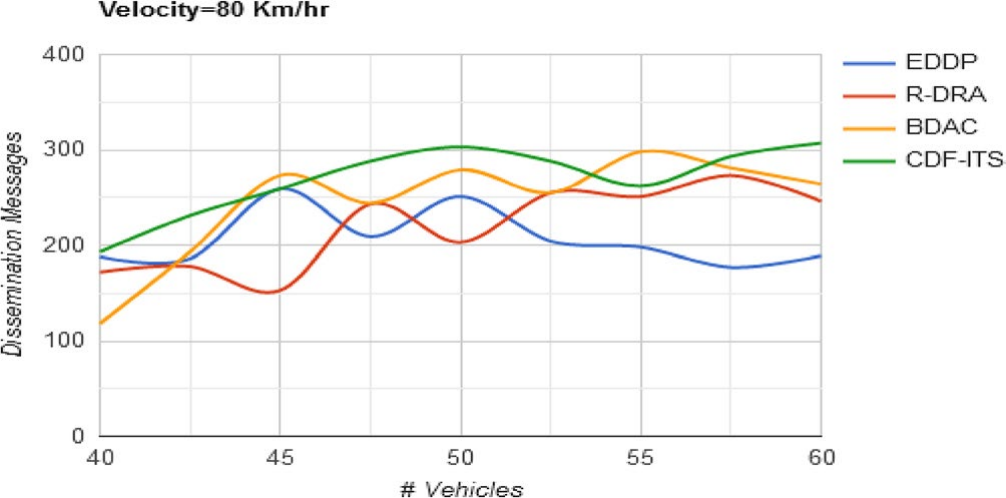
Dissemination messages versus vehicles.

The factors augmenting ${\text{max}}\left\{ \Delta \right\}$ in the varying density is the estimation of disparity that helps to approximate $f_{L}$ and $\gamma \left( {f_{L} ,\Delta } \right)$. The validation is instigated from $\Delta_{i} \ne \Delta_{i + 1}$ condition to $\frac{{C_{d} }}{{C_{r} }} < \left( {1 - f_{L} } \right)$ provided the $S$ is allocated for the requesting $v_{p}$. In such cases, the solution space determines the existence of $v_{p}$ in the dissemination process by verifying $S$ and $\varphi_{s}$. If the rate of the $\Delta$ is not below $\varphi_{s}$, the $v_{p}$ is sustained in the transmission process else, the $v_{p}$ is replaced to improve $\varphi_{s}$ to $\Delta$. This process is carried out for all the $S$ is the same manner to satisfy Eq. (2) in all $S$. Therefore, the level of $\Delta$ is retained at the least possible $\varphi_{s}$ from it is maximized to $\Delta$ (refer to Fig. 7).

**Figure 8:**
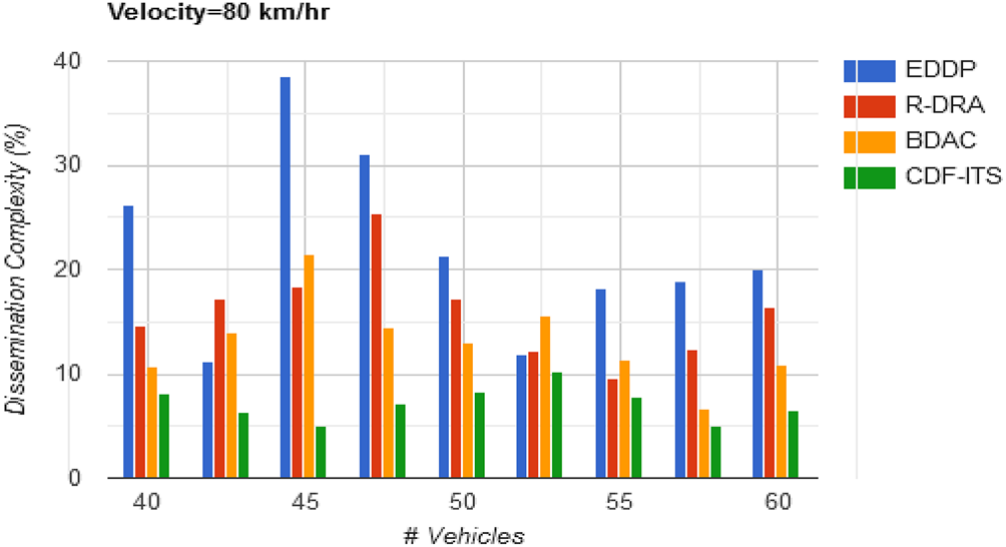
Dissemination complexity versus vehicles.

The complexity of $v_{p}$ selection is restricted until the solution space falls behind $\left[ {1 - \gamma \left( {f_{L} ,\Delta } \right)} \right]$. Whereas, the $v_{p}$ from $\left[ {1 - \gamma \left( {f_{L} ,\Delta } \right)} \right]$ to ${\text{max}}\left\{ \Delta \right\}$ region diminishes the dissemination complexity by reducing the frequency of $v_{p}$ replacement. Therefore, the need for beaconing/frequency broadcasting in the CDF is less than the existing methods in Fig. 8. Table 2 tabulates the comparative analysis result concerning the varying vehicle density.

**Table 2:** Comparative analysis with varying vehicle density (velocity = 80 km/h).

Metrics	EDDP	R-DRA	BDAC	CDF-ITS
Avg. dissemination latency (s)	1.46	1.08	0.88	0.684
Outage time (s)	0.28	0.243	0.0982	0.059
Dissemination messages	189	246	264	307
Dissemination complexity (%)	20.01	16.46	10.83	6.45

### Analysis based on varying vehicle velocity

Variation in vehicle velocity impacts the outage time of the $v_{p}$ suppose the distance is not synchronized with the $R$ of the transmitting vehicle. This caused an outage between the vehicles and demanded a recursive channel establishment process. Therefore, the complexity of content dissemination varies with the velocity. If this is not addressed, it results in transmission failures.

**Figure 9:**
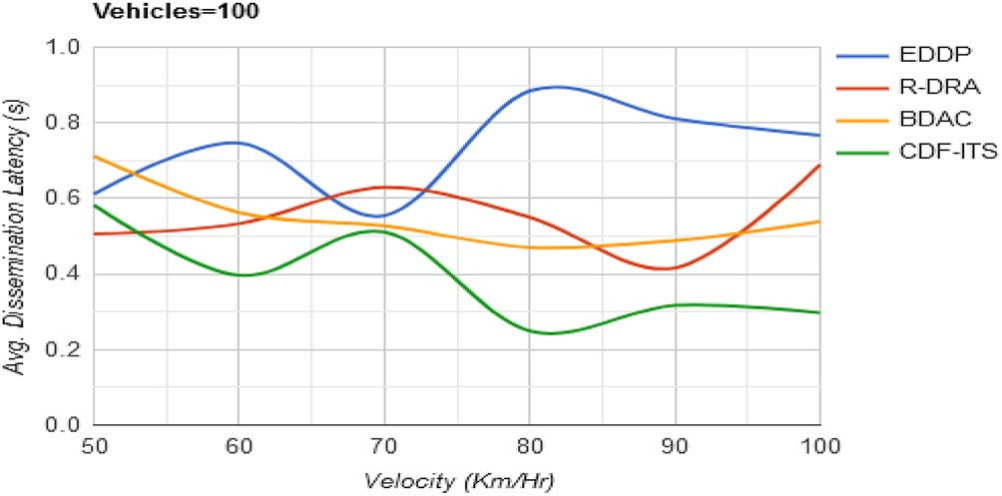
Avg. dissemination latency versus velocity.

Different from the objectives of density, outage relies on satisfying the condition of $d_{w} < d_{h}$ in all the $S$ for $n$ dissemination instances. Here, the $v_{p}$ is selected on the disparity level of $d _{w} > d_{h}$ and $d_{o} = 0$. The vehicles selected in this subspace band ensure $max\Delta$ or retain $\varphi_{s}$ post the identification of $\Delta_{i + 1}$. This case is handled for the $f_{L} \left( {d_{h} } \right)$ computed in Eq. (9b). Therefore, the $v_{p}$ satisfying the above condition and achieving ${\text{max}}\left\{ \tau \right\}$ to prevent $\Delta$ falling behind $\varphi_{s}$ ensure maximum dissemination. Hence, in this case, the dissemination on latency is $\left( {d_{h} + d_{w} } \right)$ to $\left( {d_{h} *v_{p} } \right)$, that is less compared to $\left( {d_{h} + d_{q} + d_{o} } \right)$ or $\left( {d_{h} + d_{w} } \right)*v_{p}$ (refer to Fig. 9).

**Figure 10:**
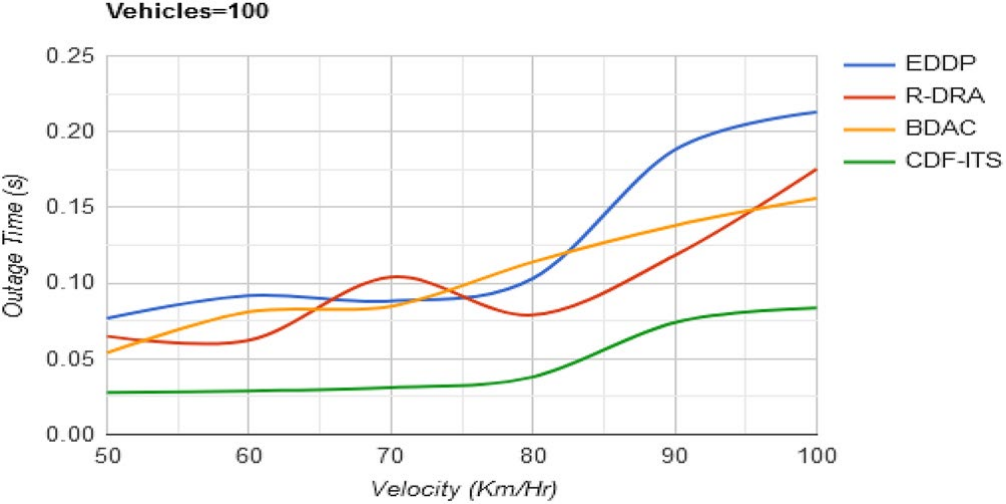
Outage time versus velocity.

The range of the solution space is confined to $\left( {d_{h} + d_{q} } \right)$ instead of $\left[ {S*\left( {d_{h} + d_{w} } \right)} \right]$ to reduce the outage in the proposed framework. The vehicles satisfying $\tau$ and $d_{o}$ constraints achieve less outage in this framework irrespective of the density of the vehicles (Refer Fig. 10).

**Figure 11:**
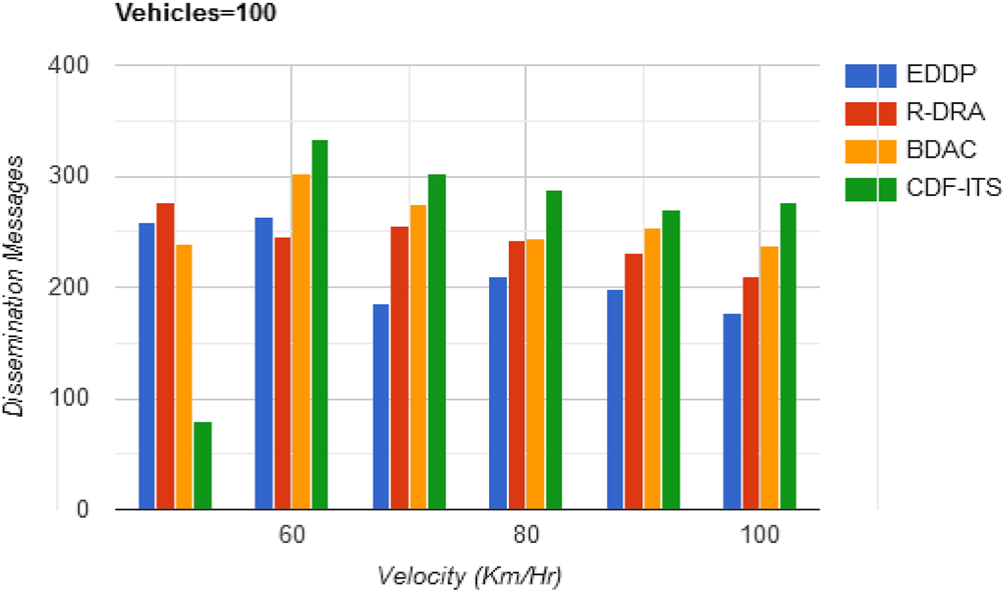
Dissemination messages versus velocity.

The channels that are less influenced by the $d_{o}$ are refined from the set of available $v_{p}$ in all the $S$ disseminations. Therefore, the rate of message handling in this case is high. Though, this rate is sustained by verifying the $\tau$ of the $v_{p}$ to achieve fairness in content dissemination. These estimated validations are required to handle the impact of the varying velocity of $^{\prime}x^{\prime}[$ in Eq. (1)] varies. This variation caused unsynchronized message dissemination, and to mitigate this impact $\tau$ based $v_{p}$ in $C$ for the allocated $S$ are opted to maximize dissemination. In such scenarios, the content dissemination (messages) is retained between $\varphi_{s}$ and $\Delta$. This achieves a high message dissemination level (refer to Fig. 11).

**Figure 12:**
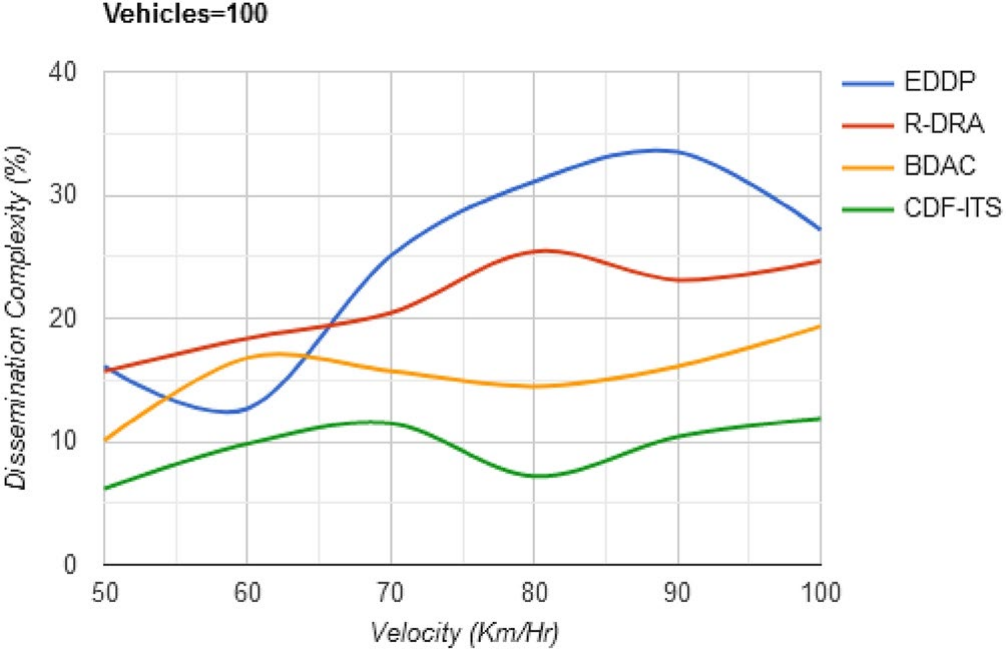
Dissemination complexity versus velocity.

The proposed CDF is different from the conventional broadcasting scheme followed in ITS. Periodic beacons for dissemination or $$v_{p}$$ selection is prevented in this framework by refining dissemination neighbours based on $$f_{L} \left( {d_{h} } \right)$$ and $$\Delta_{i}$$. The sub-optimal and disparity solution spaces are identified using decision-making that considers the variations in $$\Delta$$ in $$i$$ and $$i + 1$$ dissemination. The dissemination process is selected from the $$v_{p} \in$$ disparity, sub-optimal regions [as in Fig. 4a] require more control messages and validations compared to the solution in $$d_{w} < d_{h} )$$ and $$d_{o} = 0$$ region. If the $$v_{p}$$ lies in this space, frequent changes in dissemination path (single/multiple) are confined, augmenting $$\max \left\{ \Delta \right\}$$. Thus, the complexity due to varying velocities in dissemination is suppressed (Fig. 12). The comparative analysis results for the varying velocities are presented in Table 3.

**Table 3:** Comparative analysis for varying velocity (vehicles = 100).

Metrics	EDDP	R-DRA	BDAC	CDF-ITS
Avg. dissemination latency (s)	0.766	0.689	0.539	0.297
Outage time (s)	0.213	0.1754	0.1559	0.0837
Dissemination messages	177	209	237	276
Dissemination complexity (%)	27.18	24.63	19.37	11.87

## Conclusion

This article discusses a content delivery framework to improve ITS's message dissemination and sharing reliability. This framework employs a decision-making method that relies on the physical attributes of the vehicles to classify the dissemination feasibilities based on density and velocity. By identifying the optimal, sub-optimal, and disparity solutions, the reliable neighbour for content delivery and message dissemination is selected based on the varying features that support better delivery. The performance analysis of the proposed framework shows its reliability by improving the dissemination of messages and reducing dissemination latency, complexity, and outage. The limitations of this study include the privacy and data security concerns with robust communication systems. In the future, developments such as enhanced training of machine learning models for smart decision-making utilizing enormous training data and Big data analytics methods for higher security of smart city applications are considered.

## Data Availability

The datasets used and/or analyzed during the current study available from the corresponding author on reasonable request.

## References

[1] Dong, X. *et al.* A parallel transportation management and control system for bus rapid transit using the ACP approach. *IEEE Trans. Intell. Transp. Syst.* **18**(9), 2569–2574 (2017). 10.1109/TITS.2016.2645783

[2] Manogaran, G., Shakeel, P. M., Priyan, R. V., Chilamkurti, N. & Srivastava, A. Ant colony optimization-induced route optimization for enhancing driving range of electric vehicles. *Int. J. Commun. Syst.* **2019**, e3964. 10.1002/dac.3964 (2019). 10.1002/dac.3964

[3] Alrawi, F. The importance of intelligent transport systems in the preservation of the environment and reduction of harmful gases. *Transport. Res. Procedia* **24**, 197–203 (2017). 10.1016/j.trpro.2017.05.108

[4] Solodkiy, A. & Yenokayev, V. Cooperative ITS—a strategic way to ensure road safety. *Transport. Res. Procedia* **20**, 630–634 (2017). 10.1016/j.trpro.2017.01.102

[5] Elassali, R. *et al.* Performance evaluation of high data rate M-OAM UWB physical layer for intelligent transportation systems. *Wirel. Pers. Commun.* **94**(4), 3265–3283 (2016). 10.1007/s11277-016-3776-9

[6] Lin, Y.-W., Hsiao, Y.-K. & Yeh, Z.-S. A new mobility management scheme for intelligent transportation systems. *Wirel. Pers. Commun.* **96**(2), 3081–3112 (2017). 10.1007/s11277-017-4342-9

[7] Lin, Y.-Y. & Rubin, I. Integrated message dissemination and traffic regulation for autonomous VANETs. *IEEE Trans. Veh. Technol.* **66**(10), 8644–8658 (2017). 10.1109/TVT.2017.2700399

[8] Hanan, A. H. A., Idris, M. Y., Kaiwartya, O., Prasad, M. & Shah, R. R. Real traffic-data based evaluation of vehicular traffic environment and state-of-the-art with future issues in location-centric data dissemination for VANETs. *Digital Commun. Netw.* **3**(3), 195–210 (2017). 10.1016/j.dcan.2017.04.002

[9] Hadiwardoyo, S. A., Patra, S., Calafate, C. T., Cano, J.-C. & Manzoni, P. An intelligent transportation system application for smartphones based on vehicle position advertising and route sharing in vehicular ad-hoc networks. *J. Comput. Sci. Technol.* **33**(2), 249–262 (2018). 10.1007/s11390-018-1817-4

[10] Harrabi, S., Jaafar, I. B. & Ghedira, K. Message dissemination in vehicular networks on the basis of agent technology. *Wirel. Pers. Commun.* **96**(4), 6129–6146 (2017). 10.1007/s11277-017-4467-x

[11] Chiti, F., Fantacci, R., Gu, Y. & Han, Z. Content sharing in Internet of Vehicles: Two matching-based user-association approaches. *Veh. Commun.* **8**, 35–44 (2017).

[12] Sousa, S. *et al.* A new approach on communications architectures for intelligent transportation systems. *Procedia Comput. Sci.* **110**, 320–327 (2017). 10.1016/j.procs.2017.06.101

[13] Trullols, O., Fiore, M., Casetti, C., Chiasserini, C. & Ordinas, J. B. Planning roadside infrastructure for information dissemination in intelligent transportation systems. *Comput. Commun.* **33**(4), 432–442 (2010). 10.1016/j.comcom.2009.11.021

[14] Pan, H.-H., Wang, S.-C. & Yan, K.-Q. An integrated data exchange platform for intelligent transportation systems. *Comput. Std. Interfaces* **36**(3), 657–671 (2014). 10.1016/j.csi.2013.08.015

[15] An, C. & Wu, C. Traffic big data assisted V2X communications toward smart transportation. *Wirel. Netw.* **26**, 1601 (2019). 10.1007/s11276-019-02181-6

[16] Fan, X., Huang, C., Zhu, J. & Fu, B. R-DRA: A replication-based distributed randomized algorithm for data dissemination in connected vehicular networks. *Wirel. Netw.* **25**(7), 3767–3782 (2018). 10.1007/s11276-018-01895-3

[17] Duan, Y., Lee, V. C., Lam, K. Y., Nie, W. & Liu, K. A cross-layer design for data dissemination in vehicular ad hoc networks. *Neural Comput. Appl.* **31**(7), 2869–2887 (2019). 10.1007/s00521-017-3234-y

[18] Chaqfeh, M., El-Sayed, H. & Lakas, A. Efficient data dissemination for urban vehicular environments. *IEEE Trans. Intell. Transport. Syst.* **20**(4), 1226–1236 (2019). 10.1109/TITS.2018.2850068

[19] Zhou, Z. *et al.* Social Big-data-based content dissemination in internet of vehicles. *IEEE Trans. Ind. Inf.* **14**(2), 768–777 (2018). 10.1109/TII.2017.2733001

[20] Tiennoy, S. & Saivichit, C. Using a distributed roadside unit for the data dissemination protocol in VANET with the named data architecture. *IEEE Access* **6**, 32612–32623 (2018). 10.1109/ACCESS.2018.2840088

[21] Huang, C.-M., Lin, T.-H. & Tseng, K.-C. Data dissemination of application service by using member-centric routing protocol in a platoon of internet of vehicle (IoV). *IEEE Access* **7**, 127713–127727 (2019). 10.1109/ACCESS.2019.2936456

[22] Nguyen, T. D., Le, T.-V. & Pham, H.-A. Novel store–carry–forward scheme for message dissemination in vehicular ad-hoc networks. *ICT Express* **3**(4), 193–198 (2017). 10.1016/j.icte.2017.11.009

[23] Cao, D., Zheng, B., Ji, B., Lei, Z. & Feng, C. A robust distance-based relay selection for message dissemination in vehicular network. *Wirel. Netw.* **26**, 1755 (2018). 10.1007/s11276-018-1863-4

[24] Teng, H. *et al.* A novel code data dissemination scheme for Internet of Things through mobile vehicle of smart cities. *Future Gen. Comput. Syst.* **94**, 351–367 (2019). 10.1016/j.future.2018.11.039

[25] Baiocchi, A. Analysis of timer-based message dissemination protocols for inter-vehicle communications. *Transport. Res. Part B: Methodol.* **90**, 105–134 (2016). 10.1016/j.trb.2016.04.018

